# Blood Pressure and Blood Pressure Variability in Relation to Chronic Low Back Pain Among Patients with Hypertension

**DOI:** 10.3390/healthcare13101166

**Published:** 2025-05-16

**Authors:** Maciej Skrzypek, Michał Słaboszewski, Rafał Kolec, Wiktoria Wojciechowska, Agnieszka Olszanecka, Piotr Wróbel, Maciej Polak, Katarzyna Stolarz-Skrzypek, Marek W. Rajzer

**Affiliations:** 1 Department of Medicine and Health Sciences, Andrzej Frycz Modrzewski Krakow University, 30-705 Kraków, Poland; jester68@o2.pl (M.S.); wrobel.piotr71@wp.pl (P.W.); 2 Students Scientific Group, First Department of Cardiology, Interventional Electrocardiology and Hypertension, Faculty of Medicine, Jagiellonian University Medical College, 31-008 Kraków, Polandrafael.kolec@student.uj.edu.pl (R.K.); 3 First Department of Cardiology, Interventional Electrocardiology and Hypertension, Jagiellonian University Medical College, 30-688 Kraków, Poland; wiktoria.wojciechowska@uj.edu.pl (W.W.); agnieszka.olszanecka@uj.edu.pl (A.O.); marek.rajzer@uj.edu.pl (M.W.R.); 4 Department of Epidemiology and Population Studies, Institute of Public Health, Jagiellonian University Medical College, 31-066 Kraków, Poland; maciej.1.polak@uj.edu.pl

**Keywords:** Oswestry Disability Index, chronic low back pain, blood pressure variability, hypertension, ambulatory blood pressure

## Abstract

Introduction: Chronic pain which tends to be localised particularly in the lower back and lower extremities is one of the risk factors for elevated blood pressure (BP). In this cross-sectional study, we evaluated whether chronic low back pain (cLBP) is associated with BP variability, which may be related to increased mortality and morbidity. Methods: We included 85 consecutive hypertensive patients with a median age of 62 years (IQR, 55–67) with cLBP, for which intensity was assessed using the Oswestry Disability Index (ODI). Ambulatory blood pressure monitoring (ABPM) was performed to evaluate the values and variability of systolic blood pressure (SBP), diastolic blood pressure (DBP), and mean arterial pressure (MAP) over 24 h, day- and nighttime BP variability assessed as BP standard deviation (SD). Results: In the whole study population, the median ODI questionnaire score was 16 (IQR, 11–20). Patients with an equal/higher than median ODI score had lower nighttime DBP compared with other patients (*p* = 0.028). Equal/higher than median ODI score correlated with 24 h SD values for SBP and MAP (r = 0.263; *p* = 0.016, and r = 0.229; *p* = 0.036, respectively), as well as with day–night differences in SBP (r = 0.229; *p* = 0.035), DBP (r = 0.253; *p* = 0.019), and MAP (r = 0.263; *p* = 0.015). We performed a multivariate regression analysis adjusted for potential confounders, and equal/higher than median ODI score was predicted by age (OR, 1.07; 95% CI, 1.006–1.14; *p* = 0.031) and day–night DBP difference (OR 1.07; 95% CI 1.002–1.15; *p* = 0.044). Conclusions: To our knowledge, this is the first study to show that more intense cLBP is associated with BP variability among patients with hypertension.

## 1. Introduction

Hypertension is one of the worldwide leading risk factors for death, the prevalence of which continues to rise, with 1.56 billion people expected to be affected by 2025 [1]. Multimorbidity, a global problem, occurs in more than 50% of elderly people, and hypertension is one of the most common comorbidities [2,3]. Previous studies have suggested that chronic pain which tends to be localised particularly in the lower back and lower extremities is one of the risk factors for elevated blood pressure (BP), as 39% of patients suffering from chronic pain were diagnosed with hypertension [4]. However, physicians in primary care were 26% less likely to prescribe or intensify antihypertensive treatment among patients experiencing pain compared to those without pain complaints [5]. It has been reported that chronic pain, mostly prevalent among individuals aged 60 years or over [6], is related to excess mortality, which may be reduced by appropriate managing risk factors among those patients [7]. Interestingly, not only long-lasting pain but also BP variability may be related to increased mortality and morbidity. Both daytime and nighttime BP levels have a prognostic value in clinical practice, but it has been highlighted that 24 h ambulatory BP level remains the main predictor to be considered in risk stratification [8]. In order to measure BP, ambulatory blood pressure monitoring (ABPM) is considered a valuable method giving insight into nighttime BP parameters as well as short- and long-term BP variability [9].

We aimed to analyse the relationship between BP variability and increased Oswestry Disability Index (ODI) score in hypertensive patients. We assessed the association of pain intensity determined by the ODI score with the short- and long-term variability of BP as well as 24 h, day- and nighttime BP values.

## 2. Materials and Methods

### 2.1. Study Population

In this cross-sectional study, we investigated regular outpatients attending the hypertension clinic at the First Department of Cardiology, Interventional Electrocardiology and Hypertension, University Hospital in Krakow from May 2021 till April 2022. The hypertensive patients were eligible for the study if they were aged between 40 and 70 years old. The inclusion criteria were grade 1 or 2 hypertension according to ESC guidelines [10], regardless of treatment, and ambulatory systolic BP below 170 mmHg.

The exclusion criteria were prior myocardial infarction, coronary interventions, persistent atrial fibrillation, heart failure class II or higher according to the New York Heart Association (NYHA) classification [11], history of documented stroke or transient ischemic attacks, chronic kidney disease with estimated glomerular filtration rate (eGFR) of <30 mL/min/1.73 m^2^ at recruitment for the study.

From 134 patients who suffered from low back pain during the 3 weeks before screening, 85 consented to participate in our study. All the participants received antihypertensive treatment in the scheme of combination therapy, and none of them was on chronic painkillers. The study was conducted in line with the Declaration of Helsinki and approved by the local Bioethics Committee. All the patients gave their written informed consent.

### 2.2. ODI Questionnaire

All participants filled out an ODI questionnaire, which consists of 10 items describing the intensity of low back pain. The 10 sections cover pain intensity and daily function (including personal care, lifting objects, walking, sitting, standing, sleeping, sexual activity, social life, and travelling). Each question has six possible choices and is scored from 0 to 5. The higher score means the higher level of disability related to low back pain. The sum of the scores for all 10 questions makes for a total score range between 0 and 50. A score between 0 and 10 points is defined as minimal disability, 11 and 20 as moderate disability, 21 and 30 as severe disability, 31 and 40 as crippled, and 41 and 50 as bedridden or exaggerated [12]. Patients were divided according to the median score of the ODI questionnaire. Individuals with an equal/higher than median ODI score were defined as having a higher level of disability compared to the remainder. The ODI is an acceptable tool to measure disability related to low back pain in the general population, as its psychometric properties were well established [12]. Test–retest reliability and responsiveness were reported to be high, and the ODI showed good validity, because it was in line with other outcome measures assessing disability associated with low back pain [12]. Furthermore, it has been reported that the Polish version of the ODI is a reliable and valid instrument to measure disability among Poles [13].

### 2.3. Ambulatory Blood Pressure Monitoring

ABPM was performed in accordance with ESC/ESH guidelines [10] to assess the average values of systolic blood pressure (SBP), diastolic blood pressure (DBP), mean arterial pressure (MAP), and mean heart rate (HR) over 24 h. Measurements were performed using the oscillometric method with SpaceLabs 90207 ABP monitors (Spacelabs Healthcare, Redmond, WA, USA) with an arm cuff, which were applied to study participants on an outpatient basis. Measurements were taken at 15 min intervals during the day (from 6 a.m. to 10 p.m.) and every 30 min during the night (from 10 p.m. to 6 a.m.). Patients were instructed before the start of the measurements about their behaviour during the study. It was suggested to perform a daily activity in accordance with the patient routine with the exclusion of intensive physical exercises. Subjects were instructed to assume a position with a motionless arm at heart level while taking measurements and to keep a diary of accompanying symptoms during the study period. Measurements of SBP, DBP, and MAP were presented as mean values and median for the day, night, and full 24 h period. To correct for the number of hours included in the calculation of mean or standard deviation (SD) values for the 24 h period, the following formulas were used:

(1) $$24\;h\;BP=\frac{\left(Daytime\;BP\times 16\right)+(Nighttime\;BP\times 8)}{24}$$

(2) $$24\;h\;{SD}_{BP}=\frac{\left(Daytime\;{SD}_{BP}\times 16\right)+(Nighttime\;{SD}_{BP}\times 8)}{24}$$

where BP represents SBP, DBP, or MAP, respectively, and SD_BP_ reflects the SD of that blood pressure [8]. Day–night BP difference was defined in accordance with the following formula:

(3) $$Day\text{–}\mathrm{night}\;\mathrm{BP}\;\mathrm{difference}=\frac{Daytime\;BP-Nighttime\;BP}{Daytime\;BP}\times 100\%$$

For current analysis, we defined short-term BP variability as the SD of the BP measurements, and long-term BP variability as the day–night BP difference. Eligibility for the present analysis required an ambulatory BP recording with at least 75% of valid measurements.

### 2.4. Other Measurements

The patients completed a standardized questionnaire, and we analysed demographics, information about comorbidities, risk factors, and treatment. Weight and height were measured using standardised medical scales on an outpatient basis. Body mass index (BMI) was defined as the patient’s weight, given in kilograms, divided by the square height, given in meters. Diabetes mellitus was diagnosed if patient had prior diagnosis or when fasting glycemia was higher than 7.0 mmol/L (126 mg/dL) or glycated haemoglobin was above 6.5% [14]. Lipid-lowering therapy was coded if the patient was treated with a statin or fibrate. Hyperlipidaemia was diagnosed based on low-density lipoprotein cholesterol (LDL-C) above 2.6 mmol/L according to the 2021 European Society of Cardiology (ESC) guidelines [15] or lipid-lowering therapy. Fasting venous blood samples were taken from an antecubital vein, with the use of minimal stasis, between 8 and 11 a.m. Glucose, lipid profile and creatinine with estimated glomerular filtration rate (eGFR) were assessed by standard automated techniques.

### 2.5. Statistical Analysis

The analysis was conducted using STATISTICA 13.0 (2017; TIBCO Software Inc., Palo Alto, CA, USA). Normal distribution was checked with the use of the Shapiro–Wilk test. Continuous variables were expressed as the mean ± SD or median (interquartile range; IQR) for normal and non-normal distributions, respectively. Continuous variables were compared using Student’s *t*-test or U Mann–Whitney test, as appropriate. Categorical variables were presented as numbers (percentages) and compared by Pearson’s χ^2^ test or Fisher’s exact test. Correlations were tested using a linear Pearson correlation. Multivariable analysis was performed using logistic regression to identify factors associated with equal or higher than median ODI score. The results of the logistic regression models were presented on a forest plot, using odds ratios (ORs) with 95% confidence intervals (CIs). The discrimination of the logistic regression models was described by the area under the receiver operating characteristic (ROC) curve (AUC) with 95% CI. The significance level was set at *p* < 0.05.

## 3. Results

### 3.1. Patient Characteristics

The study sample consisted of 85 patients with a median age of 62 years (IQR, 55–67), of whom 56 (65.88%) were women. The average BMI of the study population was 28.98 (±4.34) kg/m^2^. In the whole study group, the median ODI questionnaire score was 16 (IQR, 11–20), with the lowest and highest scores in the whole study population of 1 and 46, respectively. The patients were similar in terms of demographic and clinical variables, with the exception of serum creatinine levels, which were significantly higher in males as compared to females (*p* < 0.001). The data are shown in Table 1.

Patients with equal or higher than median ODI scores presented higher BMI values and plasma creatinine concentrations as compared to the remainder, with no differences in other demographic and clinical parameters (Table 2).

### 3.2. Characteristics of ABPM Parameters by Gender

As shown in Table 3, the median of 24 h DBP was higher in males than females (*p* = 0.032). Higher nighttime DBP and nighttime MAP were also observed in men as compared to women (*p* = 0.003; *p* = 0.023, respectively). However, females differed from males in having higher day–night differences in DBP and MAP values (*p* = 0.008; *p* = 0.025, respectively).

### 3.3. ODI Score and ABPM Parameters Characteristics

The results on the relationship between ABPM parameters and ODI score showed that patients with equal/higher than median ODI score had lower nighttime DBP as compared to the remainder (*p* = 0.028). The data are shown in Table 4.

### 3.4. Associations Between ODI Score and ABPM Parameters

The results in Table 5 show a positive correlation between ODI score and 24 h SD values for SBP and MAP (r = 0.263; *p* = 0.016, and r = 0.229; *p* = 0.036, respectively). ODI score correlated inversely with nighttime DBP and MAP (r = −0.244; *p* = 0.024, and r = −0.224; *p* = 0.039, respectively). On the other hand, a positive correlation with ODI score was noted for day–night differences in SBP (r = 0.229; *p* = 0.035), DBP (r = 0.253; *p* = 0.019) and MAP (r = 0.263; *p* = 0.015).

### 3.5. Regression Analysis

We performed a multivariate regression analysis adjusted for potential confounders to find independent predictors for equal/higher than median ODI score. Each model included age, BMI, and one of the following four: nighttime SBP, nighttime DBP, day–night SBP difference, or day–night DBP difference. BMI was an independent factor for equal/higher than the median ODI score in all the presented models. After adjustment for age and BMI, lower nighttime DBP was independently associated with equal/higher than median ODI score (OR 0.95; 95% CI 0.90–0.998; *p* = 0.044). When day–night DBP difference was included in the multivariable model, equal/higher than median ODI score was predicted by age (OR, 1.07; 95% CI, 1.006–1.14; *p* = 0.031) and day–night DBP difference (OR 1.07; 95% CI 1.002–1.15; *p* = 0.044). All the models are presented in Table 6.

## 4. Discussion

To the best of our knowledge, this is the first study that analyses the association between chronic low back pain (cLBP) intensity reflected by the ODI score and the occurrence of differences in 24 h BP measurements, nighttime and daytime BP values, and short- and long-term BP variability in patients with hypertension.

The study population was representative for patients suffering from hypertension in terms of clinical and demographic characteristics [16]. As expected, men differed from women in having higher serum creatinine levels, which is consistent with the reference values shown in the previous findings [17]. According to the more numerous studies reporting BP trends with respect to gender, a significant difference in DBP and MAP, but not SBP, should be observed at a median age of ~60 years, with higher median values for males compared to females [18]. Omboni et al. showed on a group of about 53,000 subjects that day- and nighttime SBP and DBP values were higher in men as compared to women [19]. Our study showed that 24 h DBP, as well as both nighttime DBP and MAP, were higher in men compared with women. However, the nighttime SBP value tended to reach statistical significance for gender comparison, which may be due to the much smaller sample size. Moreover, what was not reported in previous studies, females had a more pronounced day–night difference in DBP and MAP as compared to males.

We observed that patients with more intense cLBP had lower nighttime DBP compared to the ones with less intense pain symptoms, and either nighttime DBP and MAP correlated inversely with ODI score. Moreover, in the multivariate analysis, not only lower nighttime DBP was an independent predictor for an equal/higher than median ODI score, but also lower nighttime SBP tended to show a similar effect. We hypothesise that the aforementioned observation may be due to the fact that patients with greater complaints could have received more care.

Our study group was representative of typical eastern European elderly patients in terms of BMI [20]. Our observations of a higher BMI in the subgroup with more intense back pain are consistent with the results of previous papers on the presence of the relationship between increased BMI and chronic pain [21]. Similarly to our observations, a study of 5058 participants showed that BMI is associated with cLBP intensity and disability [22]. However, the above-cited studies did not prove an association of this type of pain with either SBP or DBP.

Our novel finding in the hypertensive patients is the relationship between cLBP and BP variability as measured by day–night SBP, DBP, and MAP difference. A Japanese paper analysing the effect of chronic pain accompanying musculoskeletal disorders on the variability of ABP parameters showed a relationship between frailty and exacerbated SBP variations [23]. However, their study did not analyse the association with other BP parameters like DBP or MAP. A review on the relationship between posture-related, circadian, short-term, long-term BP variabilities and autonomic dysfunction in diabetes reported that such fluctuations may be a risk marker for organ damage, cardiovascular events, and mortality. Furthermore, it has been observed that such inconsistent BP values could be exacerbated by comorbidities like chronic pain [24]. A similar association was observed in a meta-analysis including 19 observational cohort studies and 17 clinical trials, in which mid- and short-term variability was linked with all-cause mortality, along with long-term BP variability, which estimated the standardised hazard ratio on cardiovascular mortality was 1.18 [25]. A Korean study suggested that nocturnal antihypertensive treatment in hypertension management has shown promise for improving BP control and reducing cardiovascular risk [26]. Hence, it is tempting to suggest that, among hypertensive individuals with chronic pain, ABP measurements and practical expedients in antihypertensive treatment should be implemented.

Several study limitations should be acknowledged. First, it was a single-centre study, but conducted in the outpatient hypertension clinic, which is a reference centre for hypertensive patients, handling large population of patients annually. Second, the sample size was relatively small, which might be a source of estimation bias and a lack of power to detect association; thus, the results obtained should be interpreted with caution. Third, daily BP variability measured by 24 h ABPM is not highly repeatable, as it has been reported that the morning surge of BP, which is responsible for day–night BP difference, is poorly reproducible, whether analysed as a categorical or continuous variable [27]. Finally, it may be challenging to detect subtle differences in disability among patients with scores near the minimum or maximum possible scale score, as the ODI questionnaire might not be sensitive enough. The ODI questionnaire does not cover all aspects of disability, as it does not include questions regarding emotional, social, and psychological factors, which may have impact on a patient’s life. Although we have adjusted our analysis for the number of covariates, we were unable to account for many factors that might influence both blood pressure and low back pain (e.g., diet, lifestyle, work/occupation).

## 5. Conclusions

In conclusion, the present study demonstrates that more intense cLBP reflected by ODI score is associated with BP variability among hypertensive patients. We suggest that pain assessment may provide additional information for patients on antihypertensive treatment. Further studies are needed to validate our observations.

## Figures and Tables

**Table 1:** Baseline patient characteristics.

Variable	All (n = 85)	Male (n = 29)	Female (n = 56)	*p* Value
Age (years)	62.00 (55.00–67.00)	61.00 (57.00–67.00)	63.00 (54.00–66.00)	0.91
BMI (kg/m^2^)	28.98 ± 4.34	28.76 ± 3.37	29.09 ± 4.79	0.74
Heart rate 24 h (beats/min)	68.00 (63.00–74.00)	69.00 (61.00–74.00)	68.00 (63.00–75.00)	0.54
Hyperlipidemia, n (%)	71 (83.53)	24 (82.76)	47 (83.93)	0.87
Lipid-lowering drugs therapy	60 (70.59)	23 (79.31)	37 (66.07)	0.31
Diabetes mellitus, n (%)	23 (27.06)	9 (31.03)	14 (25.00)	0.74
Current smoking, n (%)	11 (12.94)	2 (6.90)	9 (16.07)	0.32
Serum creatinine (μmol/L)	72.90 (65.00–86.00)	86.60 (76.00–93.00)	69.50 (62.70–78.10)	**<0.001**
eGFR (mL/min/1.73 m^2^)	80.00 (71.00–90.00)	81.00 (74.00–90.00)	78.00 (69.00–90.00)	0.30
Serum LDL-C (mmol/L)	2.33 (1.90–3.10)	2.10 (1.90–2.65)	2.50 (1.85–3.37)	0.21
NT-proBNP (pg/mL)	69.00 (38.25–118.50)	65.90 (35.50–104.00)	72.60 (39.70–186.00)	0.46

Abbreviations: BMI—body mass index; eGFR—estimated glomerular filtration rate; LDL-C—low-density lipoprotein cholesterol; NT-proBNP—N-terminal prohormone of brain natriuretic peptide. The statistical significance at which the data were bolded was 0.05.

**Table 2:** Baseline patient characteristics by median of Oswestry Disability Index score.

Variable	All (*n* = 85)	Oswestry < 16 (*n* = 37)	Oswestry ≥ 16 (*n* = 48)	*p* Value
Age (years)	62.00 (55.00–67.00)	60.00 (55.00–64.00)	64.00 (55.00–68.50)	0.11
BMI (kg/m^2^)	28.98 ± 4.34	27.86 ± 3.82	29.84 ± 4.55	**0.036**
Females, *n* (%)	56 (65.88)	21 (56.76)	35 (72.92)	0.18
Heart rate 24 h (beats/min)	68.76 ± 7.94	68.08 ± 7.40	69.29 ± 8.37	0.49
Hyperlipidemia, *n* (%)	71 (83.53)	30 (81.08)	41 (85.42)	0.81
Lipid-lowering drugs therapy	60 (70.59)	24 (64.86)	36 (75.00)	0.44
Diabetes mellitus, *n* (%)	23 (27.06)	6 (16.22)	17 (35.42)	0.08
Current smoking, *n* (%)	11 (12.94)	4 (10.81)	7 (14.58)	0.75
Alcohol intake, *n* (%)	7 (8.24)	4 (10.81)	3 (6.25)	0.69
Serum creatinine (μmol/L)	72.90 (65.00–86.80)	78.10 (69.50–92.40)	69.20 (62.30–86.00)	**0.017**
eGFR (mL/min/1.73 m^2^)	80.00 (71.00–90.00)	77.10 (71.00–88.00)	80.00 (69.00–90.00)	0.47
Serum LDL-C (mmol/L)	2.33 (1.90–3.10)	2.54 (2.10–3.00)	2.10 (1.70–3.27)	0.23

Abbreviations: BMI—body mass index; eGFR—estimated glomerular filtration rate; LDL-C—low-density lipoprotein cholesterol; NT-proBNP—N-terminal prohormone of brain natriuretic peptide. The statistical significance at which the data were bolded was 0.05.

**Table 3:** Ambulatory blood pressure parameters by gender.

Variable	Male (*n* = 29)	Female (*n* = 56)	*p* Value
24 h SBP (mm Hg)	121.00 (114.00–136.00)	118.50 (113.50–126.50)	0.15
24 h DBP (mm Hg)	73.00 (69.00–85.00)	70.00 (66.00–77.50)	**0.032**
24 h MAP (mm Hg)	90.00 (85.00–101.00)	88.00 (84.00–93.50)	0.15
24 h SBP SD (mm Hg)	13.75 (11.74–15.94)	13.50 (11.91–15.70)	0.71
24 h DBP SD (mm Hg)	9.40 (8.15–11.34)	9.49 (8.49–11.73)	0.40
24 h MAP SD (mm Hg)	10.17 (8.76–12.08)	10.43 (9.07–11.89)	0.54
Daytime SBP (mm Hg)	126.00 (118.00–138.00)	123.50 (118.00–133.00)	0.29
Daytime DBP (mm Hg)	78.00 (72.00–87.00)	75.00 (70.00–82.00)	0.12
Daytime MAP (mm Hg)	94.00 (88.00–102.00)	91.50 (87.50–85.00)	0.26
Daytime SBP SD (mm Hg)	11.73 (10.34–13.27)	11.84 (10.56–14.47)	0.61
Daytime DBP SD (mm Hg)	8.02 (6.71–9.24)	8.03 (6.85–9.87)	0.62
Daytime MAP SD (mm Hg)	9.30 (7.83–9.63)	9.07 (7.64–10.54)	0.63
Nighttime SBP (mm Hg)	115.00 (106.00–127.00)	111.00 (101.00–118.50)	0.05
Nighttime DBP (mm Hg)	68.00 (64.00–80.00)	64.00 (59.00–68.50)	**0.003**
Nighttime MAP (mm Hg)	84.00 (79.00–95.00)	80.00 (74.00–85.00)	**0.023**
Nighttime SBP SD (mm Hg)	10.79 (9.06–12.50)	11.00 (8.68–13.44)	0.95
Nighttime DBP SD (mm Hg)	7.72 (6.45–10.18)	7.53 (5.94–9.38)	0.22
Nighttime MAP SD (mm Hg)	8.25 (7.25–9.22)	8.32 (6.56–10.17)	0.81
Day–night SBP difference (%)	8.82 ± 7.88	11.31 ± 7.23	0.15
Day–night DBP difference (%)	10.49 ± 7.90	14.87 ± 6.55	**0.008**
Day–night MAP difference (%)	8.75 ± 7.54	12.40 ± 6.71	**0.025**

Abbreviations: DBP—diastolic blood pressure; MAP—mean arterial pressure; SBP—systolic blood pressure; SD—standard deviation. The statistical significance at which the data were bolded was 0.05.

**Table 4:** Ambulatory blood pressure values and variability measures by median of Oswestry Disability Index score.

Variable	All (n = 85)	Oswestry < 16 (n = 37)	Oswestry ≥ 16 (n = 48)	*p* Value
24 h SBP (mm Hg)	122.35 (114.00–129.00)	124.62 (114.00–132.00)	120.60 (113.50–127.00)	0.26
24 h DBP (mm Hg)	73.69 (67.00–79.00)	76.00 (69.00–81.00)	71.92 (67.00–76.00)	0.05
24 h MAP (mm Hg)	88.00 (84.00–94.00)	90.00 (84.00–96.00)	88.00 (83.50–93.50)	0.22
24 h SBP SD (mm Hg)	14.20 (11.88–15.73)	13.48 (11.70–15.28)	14.75 (11.91–15.94)	0.17
24 h DBP SD (mm Hg)	9.44 (8.40–11.61)	9.42 (8.32–10.92)	9.78 (8.40–12.19)	0.29
24 h MAP SD (mm Hg)	10.77 (8.85–11.90)	10.29 (8.73–11.38)	11.13 (9.19–12.51)	0.25
Daytime SBP (mm Hg)	126.91 (118.00–135.00)	128.70 (118.00–135.00)	125.52 (118.00–134.00)	0.46
Daytime DBP (mm Hg)	77.21 (71.00–82.00)	79.30 (71.00–84.00)	75.60 (70.00–81.50)	0.14
Daytime MAP (mm Hg)	92.00 (88.00–98.00)	94.00 (89.00–100.00)	91.50 (86.00–97.00)	0.36
Daytime SBP SD (mm Hg)	12.64 (10.50–14.06)	12.15 (9.74–13.16)	13.01 (10.93–14.31)	0.23
Daytime DBP SD (mm Hg)	8.35 (6.78–9.58)	8.11 (7.09–8.76)	8.54 (6.52–9.96)	0.37
Daytime MAP SD (mm Hg)	9.48 (7.79–10.47)	9.10 (7.71–10.21)	9.76 (7.87–10.53)	0.39
Nighttime SBP (mm Hg)	113.58 (102.00–121.00)	116.68 (104.00–124.00)	111.19 (101.00–118.50)	0.15
Nighttime DBP (mm Hg)	66.79 (60.00–71.00)	69.65 (61.00–75.00)	64.58 (59.00–67.00)	**0.028**
Nighttime MAP (mm Hg)	82.00 (77.00–77.00)	83.00 (78.00–92.00)	79.50 (75.00–86.00)	0.08
Nighttime SBP SD (mm Hg)	11.20 (8.87–13.27)	10.66 (8.20–13.27)	11.60 (9.17–13.29)	0.22
Nighttime DBP SD (mm Hg)	7.63 (6.23–9.77)	7.50 (6.00–9.63)	7.79 (6.38–9.84)	0.49
Nighttime MAP SD (mm Hg)	8.25 (7.02–9.83)	8.22 (6.50–9.51)	8.36 (7.21–10.04)	0.29
Day–night SBP difference (%)	10.46 ± 7.51	9.43 ± 6.94	11.25 ± 7.89	0.27
Day–night DBP difference (%)	13.37 ± 7.30	12.12 ± 7.41	14.34 ± 7.14	0.16
Day–night MAP difference (%)	11.16 ± 7.18	9.96 ± 7.03	12.08 ± 7.22	0.18

Abbreviations: DBP—diastolic blood pressure; MAP—mean arterial pressure; SBP—systolic blood pressure; SD—standard deviation. The statistical significance at which the data were bolded was 0.05.

**Table 5:** Association between Oswestry Disability Index and ambulatory blood pressure parameters.

Variable	*r*	*p* Value
24 h SBP (mm Hg)	−0.12	0.29
24 h DBP (mm Hg)	−0.16	0.15
24 h MAP (mm Hg)	−0.12	0.25
24 h SBP SD (mm Hg)	0.263	**0.016**
24 h DBP SD (mm Hg)	0.13	0.24
24 h MAP SD (mm Hg)	0.229	**0.036**
Daytime SBP (mm Hg)	−0.06	0.60
Daytime DBP (mm Hg)	−0.10	0.35
Daytime MAP (mm Hg)	−0.06	0.60
Daytime SBP SD (mm Hg)	0.16	0.15
Daytime DBP SD (mm Hg)	0.01	0.94
Daytime MAP SD (mm Hg)	0.12	0.28
Nighttime SBP (mm Hg)	−0.20	0.07
Nighttime DBP (mm Hg)	−0.244	**0.024**
Nighttime MAP (mm Hg)	−0.224	**0.039**
Nighttime SBP SD (mm Hg)	0.19	0.09
Nighttime DBP SD (mm Hg)	0.06	0.58
Nighttime MAP SD (mm Hg)	0.13	0.22
Day–night SBP difference (%)	0.229	**0.035**
Day–night DBP difference (%)	0.253	**0.019**
Day–night MAP difference (%)	0.263	**0.015**

Abbreviations: DBP—diastolic blood pressure; MAP—mean arterial pressure; SBP—systolic blood pressure; SD—standard deviation. The statistical significance at which the data were bolded was 0.05.

**Table 6:** Multivariate analysis. Logistic regression models for factors associated with equal or higher than median Oswestry Disability Index score.

	Variable	Odds Ratio	95% Confidence Interval	*p*-Value
Model 1				
	Age	1.05	0.94–1.00	0.10
	Body Mass Index	1.13	1.01–1.27	**0.032**
	Nighttime SBP	0.97	0.94–1.00	0.09
	AUC = 0.69 (0.58–0.80)			
Model 2				
	Age	1.03	0.97–1.09	0.33
	Body Mass Index	1.13	1.01–1.27	**0.039**
	Nighttime DBP	0.95	0.90–0.998	**0.044**
	AUC = 0.70 (0.58–0.81)			
Model 3				
	Age	1.06	1.003–1.13	**0.039**
	Body Mass Index	1.13	1.01–1.27	**0.035**
	Day–night SBP difference	1.06	0.99–1.13	0.11
	AUC = 0.71 (0.60–0.83)			
Model 4				
	Age	1.07	1.006–1.14	**0.031**
	Body Mass Index	1.15	1.022–1.29	**0.02**
	Day–night DBP difference	1.07	1.002–1.15	**0.044**
	AUC = 0.73 (0.62–0.84)			

Abbreviations: DBP—diastolic blood pressure; SBP—systolic blood pressure. The statistical significance at which the data were bolded was 0.05.

## Data Availability

The data presented in this study are available on request from the corresponding author due to legal restrictions, i.e., personal and medical data protection law.
